# RNA isoform diversity, splicing variants, and switching in single cells of the Alzheimer’s disease brain

**DOI:** 10.21203/rs.3.rs-7436496/v1

**Published:** 2025-09-29

**Authors:** Anis Shahnaee, Christine S. Liu, Tony Ngo, Carter R. Palmer, Derya Ziomek, Chris Park, Valerie P. Tan, Natalia L. Jimenez, Jerold Chun

**Affiliations:** 1 Center for Neurologic Diseases, Sanford Burnham Prebys Medical Discovery Institute, La Jolla, CA; 2 Contributed equally

## Abstract

Alzheimer’s disease (AD) is the most common cause of dementia, yet its molecular causes remain incompletely understood. RNA diversity in part arising from dysregulated splicing may contribute to AD pathogenesis; however, the ability to interrogate the resulting full-length isoforms in single brain cells has been technologically limited. Here we report the use of PacBio Kinnex long-read sequencing combined with 10X Genomics single-cell preparations to identify both annotated and unannotated RNA isoforms. Eight AD and seven non-diseased post-mortem human brains yielded ~70,000 single nuclei showing diverse, differentially expressed, and switched transcripts in multiple genes. Cell-type-specific isoform expression and variants with intra-exonic junctions associated with reverse transcriptase-mediated somatic gene recombination were also detected. Novel isoforms, including *CHI3L1* and *SEPTIN4,* were altered in AD. Kinnex sequencing of RNA isoforms from single nuclei detected vast isoform diversity amongst brain cell types, representing an under-explored element in AD and other brain disorders.

## INTRODUCTION

Alzheimer’s disease (AD) is the most common form of dementia^1^, currently affecting more than 30 million individuals worldwide^2^. Brain pathological features such as amyloid-beta plaques, neurofibrillary tau tangles, synaptic dysfunction, and neurodegeneration characterize AD^3–5^; however, the fundamental etiology and underlying molecular mechanisms remain incompletely known. Transcriptomic studies are providing new insights into the normal and diseased brain^6–8^, including through the use of single-nucleus RNA-seq^9, 10^ that provides cell-type level resolution^11, 12^. The vast majority of single-cell studies have utilized short-read sequencing that is suboptimal for detecting full-length mRNA isoforms, while providing an important albeit incomplete view of the transcriptome^13, 14^.

Alternative splicing leads to diversification of the transcriptome and the proteome^15, 16^. In the nervous system, the number of splice variants arising from a single gene can be extreme, as illustrated by Down syndrome cell adhesion molecule (*Dscam1* in *Drosophila*) that can be spliced into over 38,000 splice variants^17, 18^, and Neurexin 1 (*NRXN1*) in humans that can also produce thousands of isoforms^19–21^. Dysregulated splicing has been implicated in neurological disorders, including AD, ALS, and schizophrenia^22–24^. A further facet of RNA diversity first reported in the AD brain is reverse transcriptase-mediated somatic gene recombination (RT-SGR) affecting the amyloid precursor protein (*APP*) gene. Dysregulation of RT-SGR generates diverse and novel mRNAs, including those with pathogenic mutations, which can be reverse transcribed and somatically retroinserted into the genome as genomic cDNAs (gencDNAs)^25–29^. Somatic gencDNAs are analogous to germline processed pseudogenes^30–34^ but can have both coding potential and enormous sequence diversity that is generated without DNA replication (ie., somatically) within even a single individual.

Long-read sequencing enables full-length transcript identification and the classification of novel isoforms, splicing events, and disease-related transcript switching patterns^35^ and has been successfully applied to bulk tissue^36–38^. However, bulk sequencing cannot resolve cell-type-specific isoform expression, which requires single-cell approaches. Early efforts to integrate long-read sequencing technologies with single-cell profiling^29, 39, 40^ were limited by insufficient read depth. PacBio’s Kinnex^41^ overcomes this limitation through cDNA amplicon concatenation for increased sequencing depth, which was used here for the first time to investigate cell-type-resolved RNA isoform expression in single cells of the AD and non-diseased human brain.

## RESULTS

### Methodology overview and sample characteristics

**Extended Data Figure 1.**
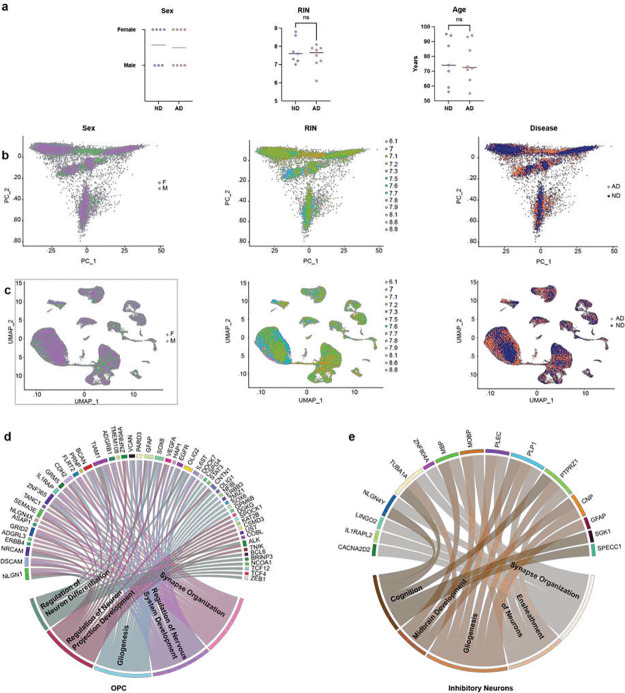
Overview of sample characteristics, covariate effects, and cell-type-specific gene ontology analysis. **(a)** Sex, RNA integrity number (RIN), and age across Alzheimer’s disease (AD) and non-disease (ND) groups (Mann–Whitney U test, P-value > 0.05). **(b)** Principal component analysis (PCA) plots colored by sex, RIN, and disease status (AD vs. ND). **(c)** UMAP visualization of samples colored by sex, RIN, and disease status. **(d)** Circos plot of differentially expressed genes in oligodendrocyte progenitor cells (OPCs), with enriched gene ontology (GO) terms and associated genes. **(e)** Circos plot of differentially expressed genes in inhibitory neurons, with corresponding GO terms and associated genes (GO term enrichments are not statistically significant; adjusted p-value > 0.05).

**Extended Data Figure 2.**
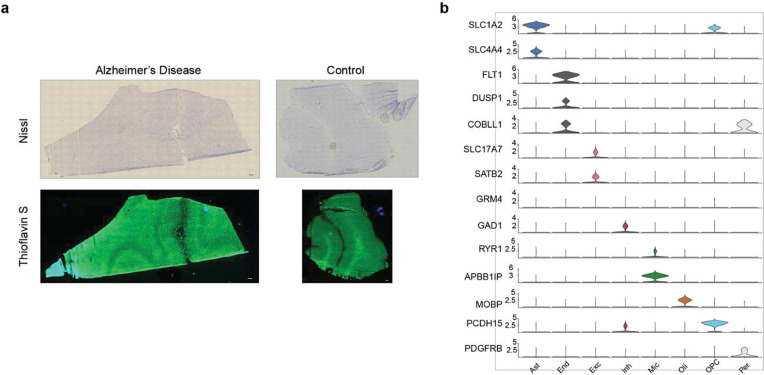
Histological validation and cell type marker gene expression. **(a)** Representative Nissl and Thioflavin S staining in AD and control brain tissues. **(b)** Violin plots indicating the expression of key marker genes used to identify cell types.

**Figure 1.**
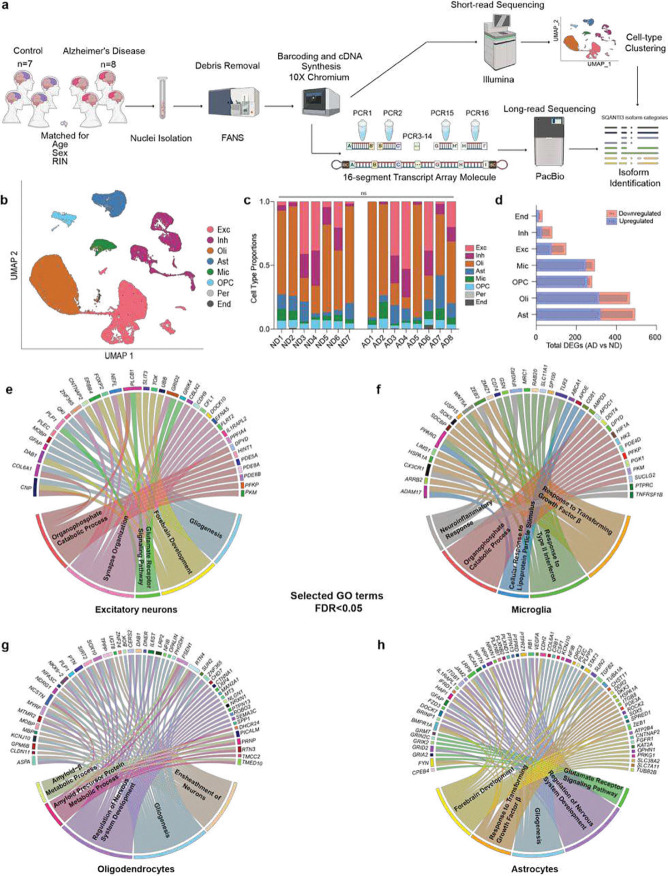
Single-nucleus short-read RNA-sequencing and Kinnex long-read sequencing workflow and cell type-specific analyses. **(a)** Schematic representation of experimental workflow for single-nucleus short-read RNA-sequencing and Kinnex long-read RNA-sequencing. **(b)** UMAP plot colored by cell type assignments. **(c)** Bar plot showing the proportions of each cell type in AD and ND samples (ns: not significant; Two-sided t-test). **(d)** Differentially expressed genes (DEGs) identified across different cell types in AD and ND samples (absolute log2 fold change > 0.25 and adjusted p-value < 0.05, Wilcoxon Rank Sum test with Bonferroni correction). **(e-h)** Circos plots of five selected Gene Ontology (GO) terms for excitatory neurons, microglia, oligodendrocytes, and astrocytes with associated genes (false discovery rate (FDR) of < 0.05 with the Benjamini-Hochberg (BH) test). Conserved GO terms are shown in the same color.

To identify isoforms and splicing alterations on a cell-type level, we performed single-nucleus short-read RNA-seq in combination with single-nucleus Kinnex long-read sequencing on prefrontal cerebral cortex tissue samples from seven non-diseased (ND) and eight Alzheimer’s disease (AD) brains (Fig. 1a). Samples were matched for age, sex, and RNA integrity number (RIN) (Mann-Whitney U test, P value>0.05) (Extended Data Fig. 1a, Supplementary Table 1). We confirmed that tissue sections contained all cortical layers using Nissl staining and that AD brains had evidence of amyloid pathology using thioflavin S staining (Extended Data Fig. 2a). Single-nucleus barcoded cDNA libraries were generated using the 10X Genomics Single Cell 3’ v3.1 kit. A portion of the amplified cDNA pool was fragmented and processed for short-read Illumina sequencing for cell type identification. To sequence full-length cDNAs, the same pre-fragmented barcoded cDNA library pool was prepared using the Kinnex method and sequenced on a PacBio Sequel II. Kinnex increases sequencing throughput by concatenating cDNA molecules to form a 16-plex array that is sequenced as a single molecule (Fig. 1a). After sequencing, the reads were then split into individual reads representing each barcoded cDNA.

### Cell-type-specific gene expression in the Alzheimer’s disease (AD) brain

Quality control filtering resulted in over 70,000 cells that were used for short-read sequencing analysis (Supplementary Table 1). Seurat (v4.3) was used to cluster cells and annotate cell types using marker genes^10^ (Extended Data Fig. 2b). All major cell types in the brain were identified, including excitatory neurons (Exc), inhibitory neurons (Inh), oligodendrocytes (Oli), astrocytes (Ast), microglia (Mic), oligodendrocyte precursor cells (OPC), pericytes (Per), and endothelial cells (End) (Fig. 1b). PCA and UMAP visualizations colored by sample-level variables - age, RIN, sex, and disease - indicated that these factors did not drive clustering (Extended Data Fig. 1b, c). No significant differences in the cell type proportions between AD and ND samples were observed (Two-sided t-test; Fig. 1c). We assessed differential gene expression in AD brains relative to ND across the major cell types, defining differentially expressed genes (DEGs) as having |log_2_ fold change| > 0.25 and a Benjamini-Hochberg corrected p-value < 0.05 (Supplementary Table 2). The number of DEGs varied in each cell type, with the highest numbers identified in astrocytes and oligodendrocytes, with 491 and 465 DEGs respectively (Fig. 1d). Gene ontology (GO) analysis^42^ revealed cell-type-specific alterations in numerous biological processes in AD brains, which were consistent with previous snRNA-seq studies^12, 43^(Fig. 1e–h, Extended Data Fig. 1d,e). For example, gene set enrichment in excitatory neurons included terms associated with “synapse organization”^12^ and “glutamate signaling” (Fig. 1e), whereas microglia showed enriched terms “neuroinflammatory response”^44^ and “response to type II interferon”, and astrocytes showed “gliosis”, all consistent with inflammatory processes in the AD brains (Fig. 1f). Oligodendrocytes showed changes to “amyloid-beta metabolic processes” (GO:0050435) and “amyloid precursor protein metabolic processes” (GO:0042982) (Fig. 1g) including alterations in *PSEN1*, providing evidence that oligodendrocytes contribute to amyloid-beta production^45, 46^. Terms related to “gliosis”, “forebrain development”, and “organophosphate catabolism” were also enriched in numerous cell types, providing evidence of widespread gene dysregulation. Importantly, these GO terms were consistent with those identified in previously published datasets^12, 43^, providing validation for the selected samples and sequencing efforts and establishing the samples as valid for parallel long-read isoform profiling.

### Isoform characteristics

**Figure 2.**
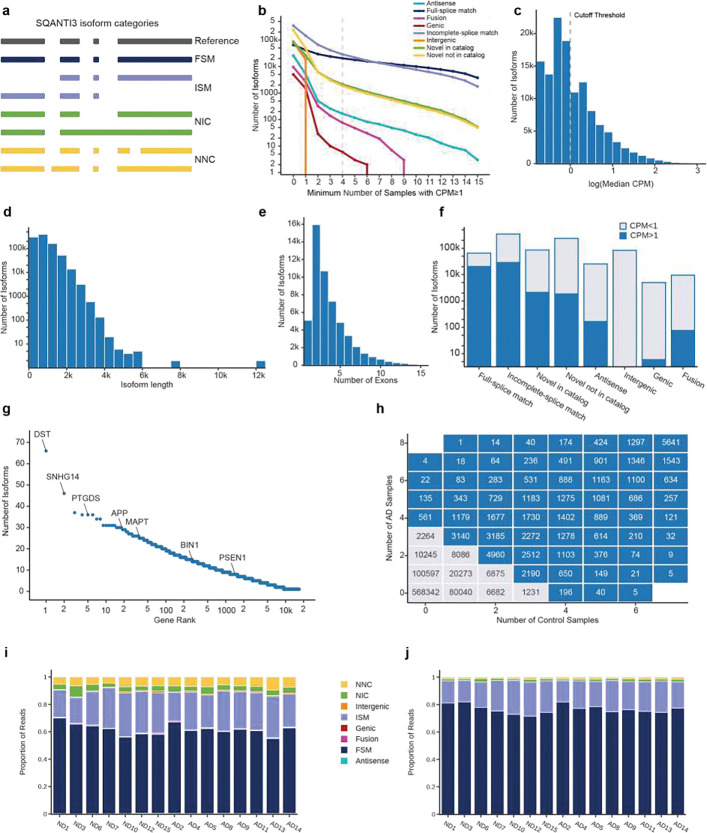
Overview of Kinnex long-read sequencing quality control and isoform filtering criteria. **(a)** Schematic representation of SQANTI3 categories used for isoform classification. **(b)** Scatter plot showing the number of isoforms with CPM ⩾1 in different numbers of samples. **(c)** Distribution of isoforms by highest CPM cutoff that would meet the four-sample cutoff. **(d)** Distribution of isoform lengths in the confident Kinnex dataset. **(e)** Bar plot showing the distribution of the number of exons in each confident isoform. **(f)** Bar plot showing the number of isoforms in each SQANTI3 category that met or did not meet the confidence cut-off. **(g)** The number of confident isoforms identified per gene (ranked by highest number of isoforms to lowest). **(h)** The number of isoforms that had CPM ⩾1 and were detected in x Control samples and y AD samples. Zero samples means that isoforms were detected with CPM <1. Gray boxes indicate isoforms that did not meet the confidence cut-off. **(i)** Bar plot showing the proportion of reads assigned to each SQANTI3 category in AD and ND samples without applying the confidence cut-off. **(j)** Bar plot showing the proportion of reads assigned to each SQANTI3 category in AD and ND samples after the confidence cut-off. These isoforms were used for further analyses.

**Figure 3.**
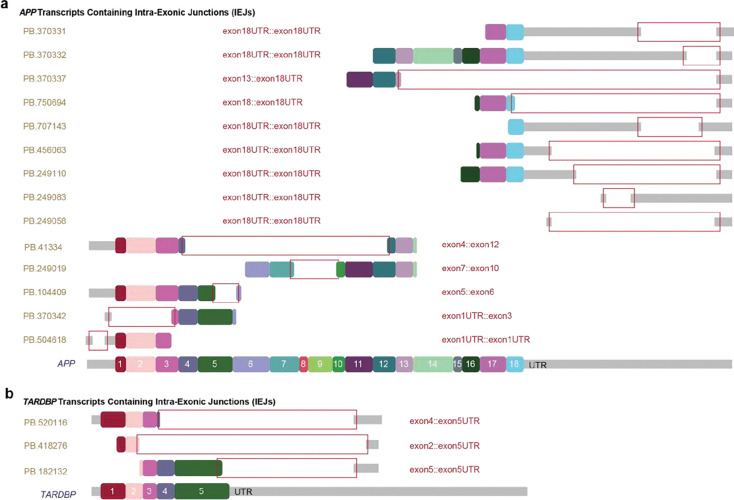
Isoforms with intra-exonic junctions **(a)** Novel *APP* transcripts containing intra-exonic junctions (IEJs, red boxes). **(b)** Novel *TARDBP* transcripts containing intra-exonic junctions (IEJs, red boxes).

A major limitation of long-read sequencing is the lower depth per sequencing run, including a higher cost per read in comparison to short-read sequencing. Kinnex improves sequencing throughput by ~16X^41^. We therefore used Kinnex and processed each sample using a complete PacBio Sequel II SMRTcell that yielded an average of 2.1 million HiFi reads (each comprising concatenated cDNAs), which were then segmented into an average of 32.5 million reads per sample (on average, 15.62 molecules per concatenated array). Reads were next processed using isoseq, a modified version of SQANTI3^39^, and isoSeQL to quantify and annotate isoforms using the GENCODE reference annotation (v44). SQANTI3 isoform categories were assigned to each of the isoforms to distinguish novel isoforms (novel in catalog, NIC; novel not in catalog, NNC) from known isoforms (full-splice match, FSM; and incomplete splice match, ISM which can be produced by incomplete reverse transcription, degradation or use of new transcription start or termination sites (TSS/TTS)) (Fig. 2a). There were 858,171 total isoforms detected (Supplementary Table 3), which were filtered to generate an operational set of “confident” isoforms detected in four or more samples with normalized expression of counts per million (CPM) ⩾ 1 (Fig. 2b, c). This confidence cutoff resulted in 53,536 confident isoforms (~6% of all detected isoforms, supported by ~75% of all reads; Supplementary Table 4). The median length of confident transcripts was 671 nucleotides long (reflecting 10X Genomics library construction limitations), and isoforms ranged from 1–31 exons (Fig. 2d, e). Novel confident isoforms were also identified (3,995 isoforms (~7% of total isoforms) comprised of 2,133 NIC isoforms and 1,862 NNC isoforms (Fig. 2f)). Annotated FSM (20,386) and ISM isoforms (28,906) were a majority of the confident isoforms, although ISM isoforms were not the focus of downstream analyses (Fig. 2f). Multiple isoforms were identified for expressed genes; notably, *DST* (dystonin gene) had the most with 66 (Fig. 2g). Inclusion of isoforms that didn’t meet the confidence cutoffs would have resulted in massive increases in diversity with an additional 83,927 NIC and 235,157 NNC isoforms supported by an average of 0.977 and 0.611 reads per sample respectively (Fig. 2h–j; Supplementary Table 3). Confidence filtering potentially ignored ~95% of the isoform diversity, some of which may arise from somatic genomic mosaicism^47^ that dilutes signals within and amongst brains through the diverse transcriptomics arising in individual cells. *APP* isoforms with intra-exonic junctions (IEJs) that can be generated by reverse transcriptase-mediated somatic gene recombination (RT-SGR) events^25, 26, 28, 29, 48^ (Fig. 3a, b) were also detected in our dataset. Isoforms with IEJs were identified for many other genes (13,618 gene/putative gene sequences; Supplementary Table 3), consistent with prior reports^26, 28, 29, 48^.

### Known and novel isoforms identified in causal and risk-factor AD genes

**Figure 4.**
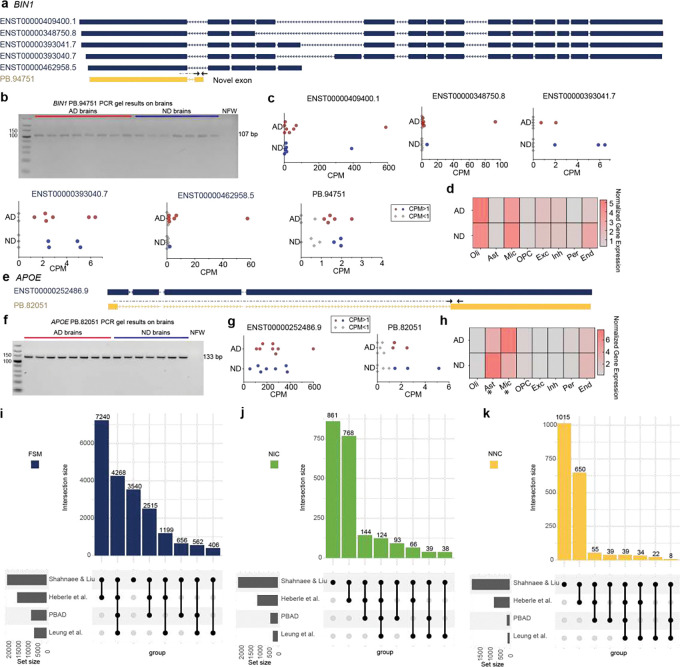
Discovery of novel transcripts of AD-related genes via Kinnex sequencing with cross-dataset comparisons. **(a)** Confident FSM *BIN1* transcripts (navy blue) and novel transcript PB.94751 (yellow). Arrows indicate the locations of the forward and reverse primers used for amplification. Dashed arrow indicates a primer that spans the exon-exon junction. **(b)** *BIN1* isoform expression in individual samples from Kinnex dataset. AD samples with CPM ⩾ 1 are represented as red circles; ND samples with CPM ⩾ 1 are represented as blue circles; and AD and ND samples with CPM < 1 are represented as grey diamonds. **(c)** Heatmap showing normalized gene expression of *BIN1* gene across different cell types from short-read sequencing data. Expression was not significantly changed between AD and ND in any cell type. **(d)** RT-PCR gel validation of the novel isoform, PB.94751. The band at 107 bp confirms that PB.94751 was detected in all 16 samples. Nuclease-free water (NFW) serves as the negative control. **(e)** Confident FSM *APOE* transcript (navy blue) and novel transcript PB.82051 (yellow). Arrows indicate the locations of the forward and reverse primers used for amplification. Dashed arrow indicates a primer that spans the exon-exon junction. **(f)** *APOE* isoform expression in individual samples from Kinnex dataset. AD samples with CPM ⩾ 1 are represented as red circles; ND samples with CPM ⩾ 1 are represented as blue circles; and AD and ND samples with CPM < 1 are represented as grey diamonds. **(g)** Normalized gene expression of *APOE* gene across different cell types from short-read sequencing data. Asterisks (*) indicate DEGs. **(h)** RT-PCR gel validation of the novel isoform, showing a band at 133 bp confirming the presence of the isoform in all 16 samples. Nuclease-free water (NFW) serves as the negative control. **(i-k)** Upset plots show FSM (i), NIC (j), and NNC (k) isoforms from our confident set detected in other datasets.

Multiple genes have been implicated causally or as risk factors in the etiology of AD. Single nucleotide mutations in *PSEN1*, *PSEN2*, and *APP*, or triplication of the AD locus, as in Down syndrome^49, 50^, cause familial forms of AD. Somatic mutations in APP have also been reported in sporadic AD^25^. Genome-wide association studies (GWAS) have identified multiple risk-factor germline gene mutations in sporadic AD. To profile how isoform expression changes could contribute to AD pathology, we interrogated multiple AD genes predicted by GWAS to contribute to AD (Supplementary Table 5). For example, several GWAS have linked mutations in *BIN1* to sporadic AD^51, 52^, and we detected five *BIN1* FSM isoforms and one NNC isoform containing a novel exon and 5’ truncation; the novel exon was validated by RT-PCR and detected in all samples (Fig. 4a,b). *BIN1* isoform expression was notably variable across samples (Fig. 4c), contrasting with results from short-read sequencing that did not report significant variability between AD and ND (Fig. 4d).

In addition to familial and GWAS AD genes, candidate gene approaches identified the well-known AD risk-factor gene *APOE* that has distinct risk levels tied to polymorphic alleles^53^. We detected two *APOE* isoforms: an FSM and a RT-PCR-validated NNC that was missing multiple exons and was expressed at significantly lower levels (Fig. 4e,f,g). *APOE* gene expression from short-read sequencing showed a distinct cell-type-specific trend across AD and ND brains, wherein AD brains had increased expression in microglia, consistent with neuroinflammation and aging^54^, but decreased expression in astrocytes (Fig. 4h). The different *APOE* gene expression patterns suggested that *APOE* and its isoforms may have distinct roles depending on cell type and disease state.

Previous long-read RNA-seq publications profiling the human brain in AD and healthy controls enabled comparison to our data^36, 37, 55^. A caveat is that our study differed from the prior studies in multiple ways: sequencing technology, library preparation method, exact neuroanatomical location, number of samples, and median sequencing depth (*i.e.,* Heberle et al: 35 million Q10 reads per sample; Leung et al: 322,000 HiFi reads per sample; PacBio: 3.9 million HiFi reads) along with known inter-individual biological variability. Published datasets were bioinformatically processed similarly to our confident set utilizing isoSeQL (Methods) and results compared to one another (Fig. 4i–k). A majority of FSM isoforms from the confident set were detected in at least one other dataset (16,846/20,386; 82.6%), with 4,268 isoforms detected in all three (Fig. 4i); 1,272 NIC (59.6%) and 847 NNC (45.5%) isoforms were also detected in at least one other dataset (Fig. 4j,k). The overlap confirmed that despite the technical and sample differences, both known and novel isoforms were consistently observed across platforms and samples. In addition, many isoforms unique to each dataset were observed as well, with 3,540 FSM, 861 NIC, and 1,015 NNC isoforms only identified in our confident isoform dataset (Fig. 4i–k).

### Differential transcript expression and switching in AD

**Figure 5.**
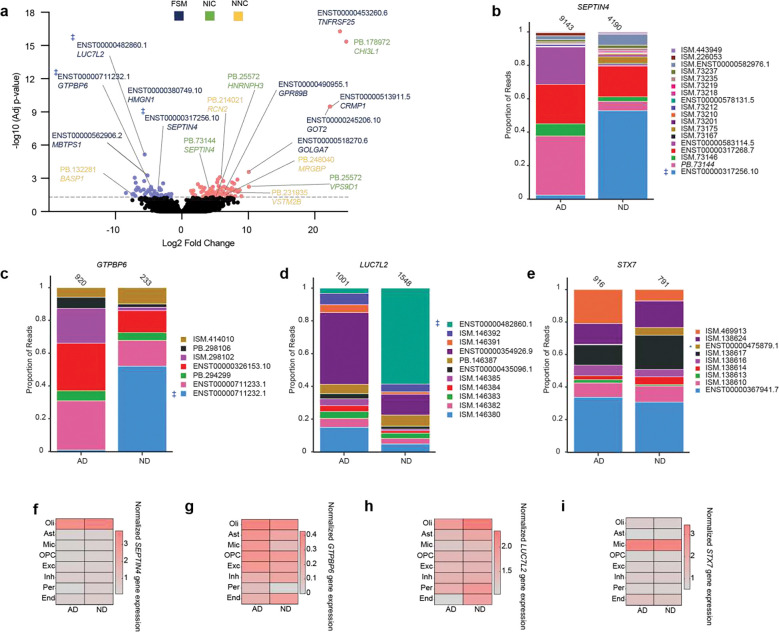
Disease-associated novel isoforms expression and transcript switching in AD. **(a)** Volcano plot of differentially expressed transcripts (DETs). Red dots represent up-regulated DETs in AD (L2FC > 1 and adjusted p-value < 0.05); blue dots represent down-regulated DETs in AD (L2FC < −1 and adjusted p-value < 0.05); and black dots indicate non-significant transcripts (adjusted p-value > 0.05). Transcripts marked with (‡) sign represent the same isoform that is both differentially expressed and switched. Isoform names are colored by SQANTI3 category: FSM (black), NIC (green), NNC (yellow) **(b-e)** Proportion of reads for each transcript in AD and ND samples. Numbers above each bar indicate total read count. **(b)** Proportion of the reads for each transcript of *SEPTIN4* gene in AD and ND samples. ENST00000317256.10 (light blue) is differentially expressed and significantly switched between ND and AD brains. **(c)** Proportion of reads for each transcript of *GTPBP6* gene in AD and ND samples. ENST00000711232.1 transcript (light blue) is differentially expressed and significantly switched between ND and AD brains. **(d)** Proportion of the reads for each transcript of *LUC7L2* gene in AD and ND samples. ENST00000482860 (teal) differentially expressed and significantly switched between ND and AD brains. **(e)** Proportion of the reads for each transcript of *STX7* gene in AD and ND samples. ENST00000475879.1 (tan) is switched between ND and AD brains. **(f-i)** Normalized gene expression across cell types from short-read sequencing data for *SEPTIN4* (f), *GTPBP6* (g), *LUC7L2* (h), and *STX7* (i).

To examine transcriptomic changes in AD, we assessed differential isoform expression and disease-related switching (via pseudobulk data analyses). Isoform switching analysis identifies the proportion of transcripts for a gene that changes with conditions, since isoform proportions can vary even if overall gene expression remains constant. Reads were aggregated by sample across all cell types, and count matrices were generated using isoSeQL for input into DESeq2 to identify differentially expressed transcripts (DETs) and DEXseq to identify switched isoforms. We identified multiple DETs in AD (Supplementary Table 6), including both known and novel isoforms (Fig. 5a), and four isoforms that were significantly switched for *GTPBP6, LUC7L2, STX7,* and *SEPTIN4* (Fig. 5b–e; Supplementary Table 7). Of the four isoforms that were switched, three were also down-regulated DETs (*GTPBP6* ENST00000711232.1, *LUC7L2* ENST00000482860.1, and *SEPTIN4* ENST00000317256.10). Interestingly, the switched isoforms’ genes were not among the DEGs detected by short-read sequencing (Fig. 5f– i).

For upregulated isoforms in AD, the most significant was an FSM of *TNFRSF25*, ENST00000453260.6 (Fig. 5a). Activation of *TNFRSF25,* also known as death receptor 3, leads to inflammation and apoptosis^56^. The upregulated isoform is truncated on the 5’ end relative to the canonical transcript, which utilizes all exons of the gene, and contains a retained intron between the sixth and seventh exons. While the final three exons are identical to the canonical transcript, the transcript is predicted to be non-coding and thus could have regulatory functions for this gene pathway. Another top upregulated DET (PB.178972) was from the gene *CHI3L1* (Fig. 5a). *CHI3L1* encodes for chitinase-3-like protein 1 (also known as YKL-40) and is a known biomarker for inflammation^57, 58^. At the transcript level, its NIC isoform (PB.178972), which has two retained introns, was significantly upregulated in AD brains (Fig. 5a), while the FSM and NNC transcripts were not differentially expressed. Consistent with our DET analysis, the FSM transcript was not detected as being expressed at different levels in AD, while the NIC isoform was significantly upregulated in AD brains (Fig. 5a)

For downregulated isoforms, multiple DETs were identified in genes that have been previously linked to brain function. The FSM isoform of *HMGN1*, ENST00000380749.10, was significantly downregulated in AD (Fig. 5a). *HMGN1* is a chromatin-binding protein involved in gene expression regulation and neural development, and altered *HMGN1* levels and function have been shown to contribute to cognitive deficits in Down syndrome^59, 60^. A novel isoform of *BASP1* was also found to be downregulated in AD. BASP1 is a signaling protein involved in neurite outgrowth and synaptic plasticity^61^ that has been explored as a potential CSF biomarker for Alzheimer’s disease^62^. The *BASP1* NNC isoform (PB.132281) contained a novel exon on the 5’ end. Its predicted protein sequence matches previously annotated *BASP1* isoforms, ENST00000322611.4 and ENST00000616743.1, suggesting that the novel exon serves as an alternate 5’UTR that may affect its stability and translation efficiency.

**Figure 6.**
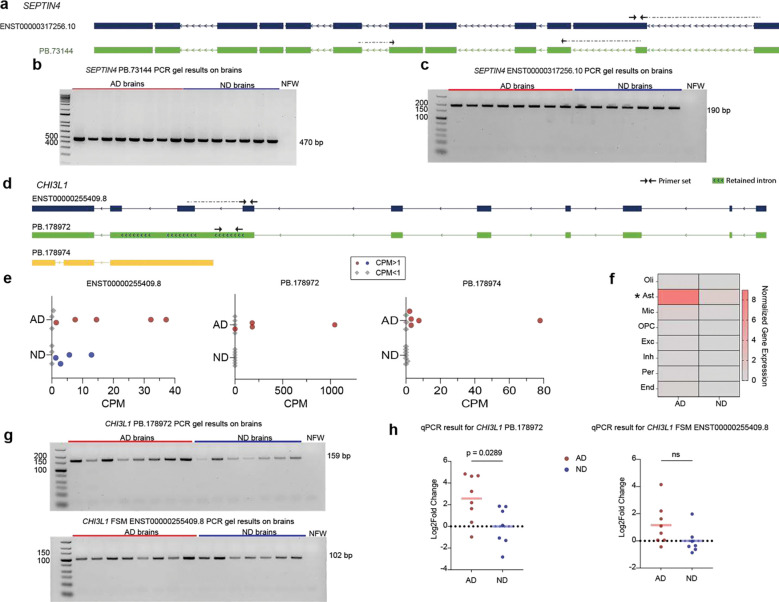
DET isoforms expression. **(a)** Confident FSM isoform (navy blue) and NIC isoform (green) of *SEPTIN4*. Arrows indicate forward and reverse primers. Dashed arrows indicate primers spanning an exon-exon junction. **(b,c)** Gel electrophoresis validation of RT-PCR for *SEPTIN4* PB.73144 NIC isoform (b) and *SEPTIN4 ENST00000317256.10* isoform (c). Nuclease-free water (NFW) was used as a negative control. **(d)** Confident FSM isoform (navy blue), NIC isoform (green), and NNC isoform (yellow) of *CHI3L1*. Arrows indicate forward and reverse primers. Dashed arrows indicate primers spanning an exon-exon junction. **(e)** *CHI3L1* isoform expression in individual samples from Kinnex dataset. AD samples with CPM ⩾ 1 are represented as red circles; ND samples with CPM ⩾ 1 are represented as blue circles; and AD and ND samples with CPM < 1 are represented as grey diamonds. **(g)** Heatmap showing normalized gene expression of *CHI3L1* gene across different cell types from short-read sequencing data. Asterisk (*) indicate DEGs in Astrocytes. **(h)** Gel electrophoresis validation of RT-PCR for *CHI3L1* PB.178972 NIC isoform and *CHI3L1 ENST00000255409.8* isoform **(i)**. Nuclease-free water (NFW) was used as a negative control. **(j)** RT-qPCR validation of the FSM and NIC transcript. NIC was significantly upregulated in AD brains (p-value: 0.0289; Mann-Whitney U test, (two-tailed)), whereas FSM isoform showed no difference (not significant: ns= 0.0721).

*SEPTIN4* had multiple DETs in AD including a down-regulated and significantly switched FSM isoform (ENST00000317256.10) and an up-regulated NIC isoform (PB.73144) with part of exon 2 missing (Fig. 6a), both of which were validated by RT-PCR (Fig. 6b, c). *SEPTIN4* encodes a GTP-binding cytoskeletal protein involved in neuronal structure and synaptic function^63^. It has also been found in pathological protein aggregates of both Parkinson’s disease and AD^64, 65^. Another gene expressed by reactive astrocytes^66^ was *CHI3L1* that encodes YKL-40 and is found at higher levels in the cerebrospinal fluid, positively correlating with disease severity and cognitive decline in AD^57^. We identified three *CHI3L1* transcripts (Fig. 6d) with varying expression across AD and ND brains (Fig. 6e). Based on gene-level analysis from short-read sequencing, *CHI3L1* was significantly upregulated in AD astrocytes and had limited expression in other cell types (Fig. 6f). Both FSM and NIC transcripts were validated by RT-PCR (Fig. 6g). Consistent with our DET analysis, the FSM transcript was not expressed at different levels in AD compared to ND, while the NIC isoform was significantly upregulated in AD brains (Fig. 6h). Upregulation of *CHI3L1* observed at the gene level is likely driven by the increased expression of isoform PB.178972, given that the other isoforms were minimally expressed in comparison and did not change in expression in AD.

### Distinct transcript expression patterns across cell types

**Figure 7.**
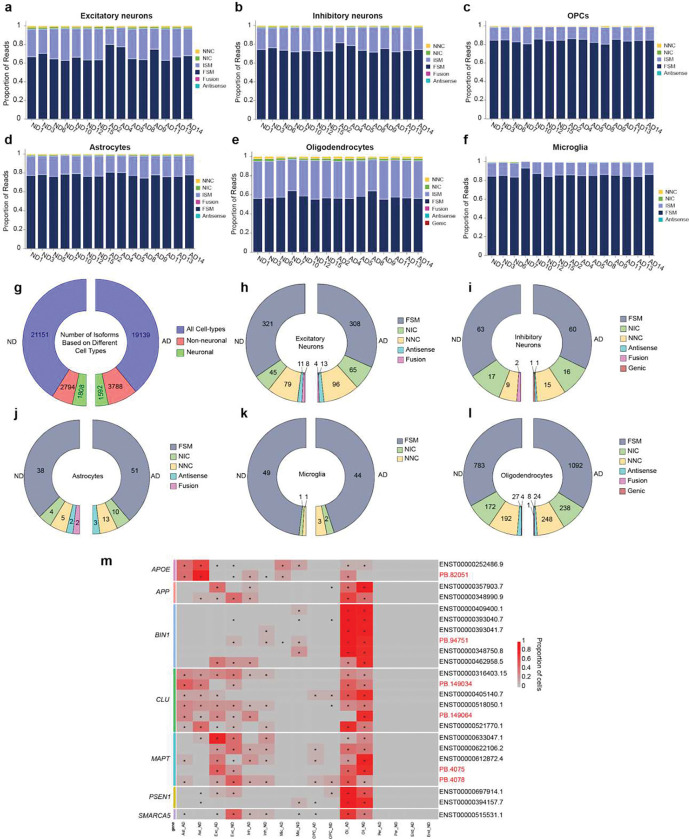
Isoform expression pattern across cell-types in AD and ND brains. **(a-f)** Proportions of reads for different isoform categories (FSM, ISM, NIC, NNC, genic, antisense, and fusion) across different cell types in AD and ND. **(g)** Number of isoforms detected in each cell type, separated by neuronal (excitatory and inhibitory neurons) and non-neuronal (astrocytes, microglia, oligodendrocytes, and OPC) and shared across the neuronal and non-neuronal, separated by AD and ND brains. **(h-l)** Number of unique isoforms expressed in only one cell type, suggesting transcript specificity across cell types. **(m)** Heatmap showing cell-type proportion in which each isoform was detected from neurological genes across neurons and glia, separated by AD and ND brains. * indicates cell types that made up 5% of the total cells in which the isoform was detected.

Cell type assignments for isoforms were made by using short-read analysis by employing libraries sharing the same cellular barcodes (Supplementary Table 8). A transcript was determined to be expressed in a particular cell type if that cell type made up at least 5% of the cells in which the isoform was detected. In all cell types, FSM isoforms comprised a higher proportion of reads, while NIC and NNC had the lowest read proportions (excluding fusion, antisense, and genic sequences) (Fig. 7a–f). In both AD and ND, most isoforms were present in neuronal and non-neuronal cell types (Fig. 7g). Also, while most isoforms were expressed in more than one cell type, each of the major cell types possessed a unique set of isoforms, suggesting specialized functions for transcript isoforms within that cell type population (Fig. 7g–l).

**Figure 8.**
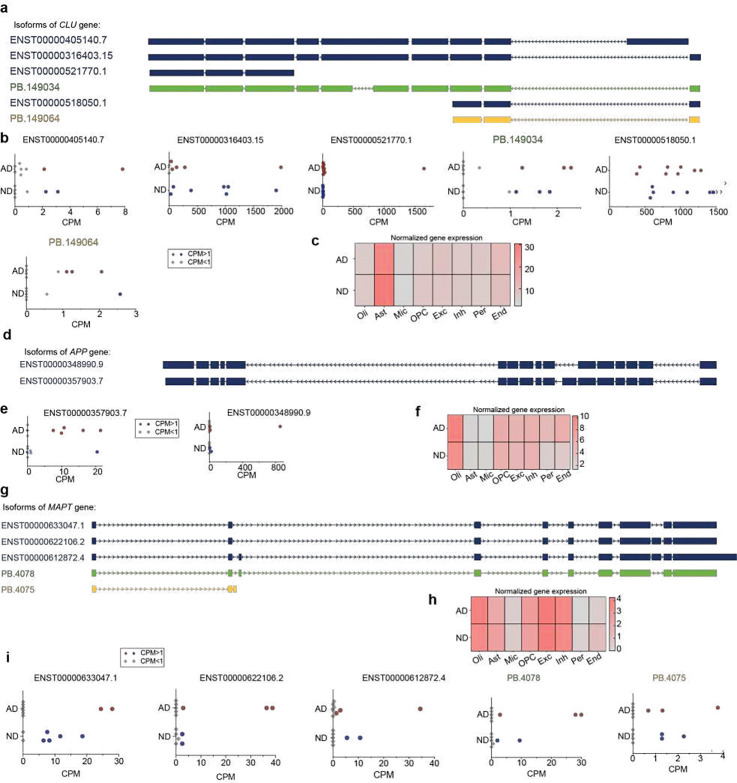
Transcripts of AD-related genes. **(a)** Confident FSM *CLU* transcripts (navy blue), PB.149034 (green) NIC isoform, and PB.149064 (yellow) NNC isoform. **(b)** *CLU* isoform expression in individual samples from Kinnex dataset. AD samples with CPM ⩾ 1 are represented as red circles; ND samples with CPM ⩾ 1 are represented as blue circles; and AD and ND samples with CPM < 1 are represented as grey diamonds. **(c)** Heatmap showing normalized gene expression of *CLU* gene across different cell types from short-read sequencing data. Expression was not significantly changed between AD and ND in any cell type. **(d)** Confident FSM *APP* transcripts (navy blue). **(e)** *APP* isoform expression in individual samples from Kinnex dataset. AD samples with CPM ⩾ 1 are represented as red circles; ND samples with CPM ⩾ 1 are represented as blue circles; and AD and ND samples with CPM < 1 are represented as grey diamonds. **(f)** Heatmap showing normalized gene expression of *APP* gene across different cell types from short-read sequencing data. Expression was not significantly changed between AD and ND in any cell type. **(g)** Confident FSM *MAPT* transcripts (navy blue), PB.4087 NIC isoform (green), and PB.4075 NNC isoform (yellow). **(h)** Heatmap showing normalized gene expression of *MAPT* gene across different cell types from short-read sequencing data. Expression was not significantly changed between AD and ND in any cell type. **(i)** *MAPT* isoform expression in individual samples from Kinnex dataset. AD samples with CPM ⩾ 1 are represented as red circles; ND samples with CPM ⩾ 1 are represented as blue circles; and AD and ND samples with CPM < 1 are represented as grey diamonds.

**Figure 9.**
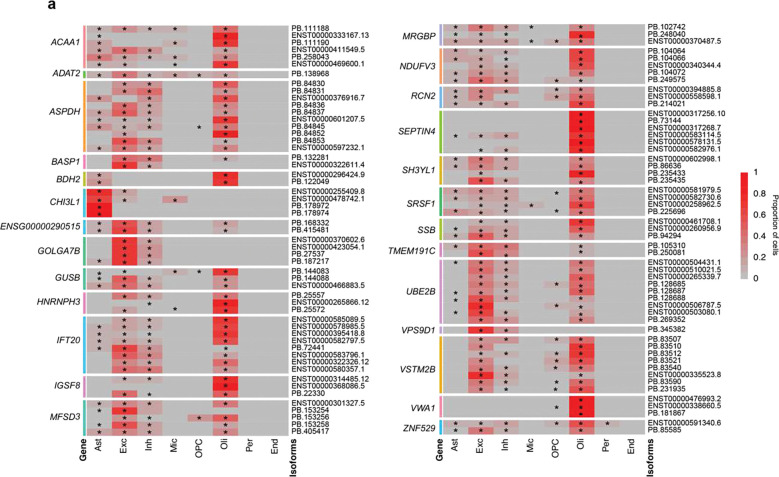
DET Isoform expression pattern across cell-types in AD and ND brains. **(a)** Heatmap showing proportion of cells in which each isoform was detected from DET genes across neurons and glia. * indicates cell types that made up 5% of the total cells in which the isoform was detected.

A focused examination of six AD-related genes – *APOE, APP, BIN1, CLU, MAPT* and *PSEN1* – and *SMARCA5*, which has been linked to neurological disorders, revealed different expression patterns across cell types and AD (Fig. 7m). For example, a novel *APOE* isoform (PB.82051; Fig. 4e) was not detected in oligodendrocytes of ND brains. In contrast, the *CLU* PB.149064 isoform (Fig. 7m, Fig. 8a–c) was not detected in oligodendrocytes of AD brains. Two different *APP* isoforms (ENST00000348990.9/APP-695, ENST00000357903.7/APP-751, Fig. 8d) with varying expression across AD and ND brains but not among DEGs (Fig. 8e,f), were detected in inhibitory neurons of AD brains but were absent from ND samples. Moreover, the APP-695 isoform was predominantly expressed in AD excitatory neurons, whereas the APP-751 isoform was predominantly in ND excitatory neurons (Fig. 7m). In contrast, an inverse pattern was observed in oligodendrocytes (Fig. 7m). Two novel isoforms of *MAPT* (PB.4075 and PB.4078, Fig. 8g–i) were both detected in excitatory neurons whereas only one, PB.4078, was found in inhibitory neurons. Furthermore, other genes beyond the AD set had expression patterns that varied by cell type. Genes with DETs identified from the pseudobulk analysis displayed differing patterns with some isoforms detected in single cell types while others showed widespread expression (Fig. 9a). For example, *CHI3L1* PB.178972 and PB.178974 (Fig. 6d) was detected only in astrocytes (Fig. 9a), and *SEPTIN4* PB.73144 only in oligodendrocytes (Fig. 9a). Overall, these isoform data complement and augment prior short-read transcriptomic studies and demonstrate isoform diversity varying amongst cell types and AD state.

## DISCUSSION

RNA sequence diversity generated by mechanisms that include splicing has been implicated in AD, however its range of isoforms and relationships to specific cell types and disease remain unclear and/or unassessed by prior short-read single-cell sequencing approaches. Long-read sequencing studies have implicated RNA diversity in single brain cells, albeit hampered by limited sequencing depth^29^. PacBio Kinnex addresses this limitation by increasing long-read sequencing depth by ~16X through template concatemerization. Its application here to single-nucleus RNA-sequencing from human brain revealed a vast level of diverse RNA isoforms, ranging from known splice variants albeit not previously linked to specific cell types, to unknown transcripts. Differential transcript expression and switching analysis of pseudobulk isoforms revealed isoform changes that would be overlooked by only short-read methods. Limitations not unique to this study included, sample size for disease vs. non-disease comparisons, 10X library preparations that limit cDNA length, along with overall cost. Other considerations of this study that warrant further detail are discussed below.

The use of nuclei rather than whole cells is standard for single-cell techniques to study human brain tissue^9, 10^, an approach that has been shown to be representative of gene expression in the cytosol^67, 68^. However, as with bulk long-read sequencing of total RNA, concerns arise regarding the inclusion of premature mRNA isoforms. Other groups have tried to limit the capture of premature mRNA through the use of exome enrichments with probes targeting exonic sequences^69^. We opted for a filtering approach, assuming that partially spliced or fully unspliced transcripts would only be present transiently and should not be detected consistently across multiple samples. We note that transcripts with retained introns are part of reference annotations (GENCODE/Ensembl), and that intron retention is not only a feature of premature mRNA, but also a result of alternative splicing.

Filtering transcriptomic data is essential for distinguishing biologically meaningful isoforms from technical artifacts^36^. We chose a confidence cutoff to generate a set of high-quality isoforms that we could compare between our AD and ND groups in addition to other studies, assuming that confident transcripts would be identified more consistently across multiple samples than artifacts of library preparation and sequencing. Somatic mosaicism, however, may introduce isoform diversity consistent with the brain’s vast genomic, inter-individual heterogeneity that is excluded using such filtering methods^47, 70, 71^. RT-SGR of genes within single neurons produces additional copies of a gene that, if transcribed, may only be expressed in a few cells or even a single cell within an individual brain^25, 72^. As an example, several isoforms of *SMARCA5* did not meet confidence cutoffs but may be indicative of their potential to undergo RT-SGR (Supplementary Table 3). Future analyses will be needed to identify and interpret the many isoforms removed in the filtering process.

Disease-related transcript differences were observed for many genes, with specific isoforms being expressed in defined cell types. Two notable examples that showed AD-dependent changes in specific RNA isoforms were *SEPTIN4* and *CHI3L1,* both of which highlight the importance of examining isoform-level expression. *SEPTIN4* was not a DEG (differentially expressed gene) in any cell type using short-read RNA-seq, likely explained by opposing changes in two isoforms – one upregulated, the other downregulated – that masked detection of differential expression at the gene level, highlighting the need for isoform resolution. *CHI3L1* was differentially expressed at the gene level in astrocytes, however our analysis revealed that most of its expression could be attributed to a novel isoform (PB.178972) rather than the known transcript. This novel transcript itself was significantly upregulated in AD, with no change detected in the expression of the known isoform, as validated with RT-qPCR. Given these isoforms’ disease-associated expression patterns, they may represent novel mechanisms in AD and/or useful biomarker candidates, but require further study.

This study highlights the advantages of long-read RNA sequencing for resolving transcript-level changes that are often obscured in gene-level analyses of single brain cells. Different isoforms of the same gene can have distinct, and sometimes opposing, expression patterns, underscoring a need to capture isoforms to better understand the molecular complexities of the brain and diseases like AD. Future studies integrating long-read sequencing with functional assays will be essential to further characterize isoforms’ relevance to disease mechanisms and their potential uses as biomarkers or targets for therapeutic intervention.

## METHODS

### Tissue selection and preparation

Fifteen prefrontal cortices were selected from various brain bank sources (8 female and 7 male donors. The sources are: Emory University (n=9), UK Imperial (n=3), Southwest Neurodegenerative Diseases Brain Bank (n=2), Pittsburgh Neurodegenerative Diseases Brain Bank (n=1), and Sepulveda Neurodegenerative Diseases Brain Bank (n=1). Samples were transferred from −80 °C freezers to a cryostat set at −18 °C. The samples were sectioned serially to obtain 200–300 μm sections for nuclei isolation and 20 μm sections for RNA integrity number (RIN) value analysis, as well as Nissl, Thioflavin S staining, RT-PCR, and RT-qPCR. Samples were matched for age, sex and RIN (Mann-Whitney U test).

### Nissl staining

Brain sections (20 μm) were mounted on glass slides [Fisherbrand^™^ Tissue Path Superfrost^™^ Plus Gold Slides, 22–035813] and fixed in 10% neutral buffered formalin (NBF) [Epredia, 22–050-104] for 5 minutes at room temperature. Sections were incubated in 70% and 95% ethanol for 3 minutes each, then 100% ethanol [Decon^™^ Labs, 04–355-222] for 20 minutes, followed by rehydration steps in 95% and 70% ethanol, and rinsed in deionized (DI) water. Sections were incubated in 0.2 % Cresyl violet solution for 5 minutes. Excess stain was removed with DI water (3 × 30 seconds), 70% ethanol and 95% ethanol for 1 minute each, and xylene [Fisher Chemical, X5SK-4] for 3 minutes. Coverslips were used with Cytoseal 60 mounting medium [Epredia, 23–244257] to seal the slides.

### Thioflavin S staining

Brain sections (20 μm) were mounted on glass slides and fixed in 10% NBF for 5 minutes at room temperature. Sections were washed with DI water twice and then incubated in 1% Thioflavin S solution for 8 minutes. Sections were washed again with DI water and counterstained with 5 μg/mL DAPI [Sigma-Aldrich, D9542–5MG] for 3 minutes at room temperature, followed by a DI water rinse. Sections were dehydrated in 70% ethanol for 4 minutes, 95% ethanol for 3 minutes, 100% ethanol for 1 minute, and then incubated in xylene for 3 minutes. Coverslips were used with Cytoseal 60 mounting medium to seal the slides.

### RIN measurement

RNA was isolated from 20 μm sectioned brain samples using the RNeasy Mini Kit [Qiagen, 74106]. Samples were then diluted to 3 ng/μL, and their RIN values were measured on an Agilent 4200 TapeStation using Agilent High Sensitivity RNA ScreenTape at the Sanford Burnham Prebys Genomics Core. Samples with RIN ≥ 6 were included for further analysis (Supplementary Table 1).

### Nuclei isolation

Human postmortem brain tissues were randomly sorted into four groups for processing to negate potential batch effects. To each sample, 1 mL of chilled nuclei buffer (250 mM sucrose [Fisher Chemical, S3–212], 1 mM DTT [Invitrogen, D1532], 10 mM Tris-HCl pH 7.5 [Invitrogen, 15567], 25 mM KCl [Sigma-Aldrich, P9333], 5 mM MgCl_2_·6H_2_O [Sigma-Aldrich, M9272], 0.1% Triton X-100 [Sigma-Aldrich, T8787], 1 U/μL Protector RNase Inhibitor [Millipore Sigma, 3335402001], and cOmplete EDTA-free protease inhibitors [Roche, 11836170001]) were added and incubated for 5 minutes. Samples were gently homogenized using a tissue grinder [Wheaton, 358005] with 20 strokes. A 50 μm cell filter [Sysmex, 04–0042-2317] was used to filter the homogenate and remove debris. The samples were then centrifuged at 820 RCF for 5 minutes at 4 °C. The supernatant was aspirated, and the pellet was resuspended in 500 μL of cold sorting buffer (PBS with 10% BSA [Gemini Bioproducts, 700–107P], 5 μg/mL 7-AAD [Sigma, A9400], cOmplete EDTA-free protease inhibitors, and Protector RNase Inhibitor) and mixed gently five times. Fluorescence-activated nuclear sorting (FANS) was performed using a FACSAria Fusion [BD Biosciences] by gating out debris and selecting 7-AAD+ singlets to isolate an average of 200,000 nuclei per sample. Following sorting, nuclei were resuspended in PBS with 10% BSA and 5 U/μL Protector RNase Inhibitor, then diluted to a final concentration of 200–1,200 nuclei/mL.

### 10x Genomics 3’ cDNA library generation

To generate 3′ cDNA libraries, single-nuclei suspensions were processed using the Chromium Next GEM Single Cell 3′ Reagent Kit v3.1 (Dual Index), according to the manufacturer’s protocol (10x Genomics). From each sample, 7,000–8,000 nuclei were encapsulated into Gel Beads-in-Emulsion (GEMs) using the Chromium Controller, and the RT reaction took place. Barcoded full-length cDNA was purified using Dynabeads^™^ MyOne^™^ SILANE. The purified cDNA was then amplified by PCR. The pre-fragmented cDNA library was split between Illumina NovaSeq 6000 short-read (25%) and PacBio Sequel II long-read sequencing (75%).

### 3’ cDNA library fragmentation for Illumina short-read sequencing

Amplified cDNA (25%) was fragmented using the fragmentation enzyme and fragmentation buffer provided in the Chromium Library Construction Kit. End-repair and A-tailing were performed after fragmentation. Sequencing adaptors and Illumina indices were ligated to the DNA fragments. The final libraries were purified, and the quality and size distribution were assessed using an Agilent Fragment Analyzer. The generated cDNA libraries were sent to Azenta/Genewiz for sequencing on the NovaSeq 6000 for an average of 62,612 reads per cell.

### Kinnex library preparation and sequencing

Pre-fragmented cDNA libraries (75%) were used for MAS-Seq/Kinnex sequencing using “MAS-Seq for 10x Single Cell 3′ kit” (PacBio) according to the manufacturer’s instructions. Before starting the MAS/Kinnex protocol, the cDNA libraries underwent a quality control step to evaluate size distribution and quantity using a High Sensitivity DNA kit by Agilent Femto Pulse (FP-1101–0275). A PCR step was then performed to add biotin tags to DNA fragments, enabling the removal of template switching oligos (TSOs) that were generated during cDNA synthesis in the 10x protocol. In the next step, cDNA libraries were amplified in 16 separate PCR reactions using various primers (A–P MAS primer mix). The PCR-amplified fragments were then concatenated into a longer concatemer called the MAS array. DNA damage repair, nuclease treatment, and a final clean-up with SMRTbell beads were performed at the end of the library preparation. All final SMRTbell libraries were quality controlled using the Agilent Femto Pulse instrument.

Each sample was loaded onto its own 8M SMRTCell and sequenced on the PacBio Sequel II. On average 2,077,210 HiFi reads were obtained per sample.

### Short-read snRNA-seq analysis

#### Processing/QC

Using 10x Genomics Cell Ranger (v7.1.0), samples were demultiplexed, and reads were aligned to the reference genome to quantify unique molecular identifiers (UMIs) and create a cell-count matrix. Because sequencing libraries were prepared from isolated nuclei and not whole cells, a pre-mRNA reference file (ENSEMBL GRCh38) was used with the default parameters to capture intronic reads originating from nuclear pre-mRNAs. Seurat (v4.3) was used to process these cell-count matrices. Nuclei were removed if they expressed fewer than 300 genes, contained greater than 1% of reads mapping to mitochondrial genes, or exceeded an outlier cutoff of UMIs from interquartile range calculations (to remove potential doublets). Each sample was normalized using Seurat’s SCTransform() function with vst.flavor=”v2” to prepare for integration.

#### Cell type identification and differentially expressed gene analysis

SCTransform()-normalized datasets were integrated into a single Seurat object following the recommended workflow using PrepSCTIntegration(), FindIntegrationAnchors(), and IntegrateData(). To identify cell types, labels were transferred from a reference dataset generated from human cortical samples (Lake et al., 2018) using TransferData() (dims=1:30). UMAP embeddings were generated (1:50), and clusters were generated using FindNeighbors() (dims=1:50) and FindClusters() (res=0.3). Differentially expressed genes between cell types in Alzheimer’s disease and in non-diseased sample groups were identified using FindMarkers() with default parameters. Genes were labeled as DEGs if |log2fold|>0.25 and the adjusted p-value <0.05 using the Wilcoxon Rank Sum test with Bonferroni correction.

### Gene ontology (GO) analysis

GO analysis was performed using WebGestalt. In the basic parameters, overrepresentation analysis as the method of interest, *Homo sapiens* as the organism of interest, and GO with the subcategory ‘Biological Process (no redundant)’ were selected, and the analysis type was set to gene/protein. Cell-type-specific DEGs comparing diseased to non-diseased samples were uploaded. A false discovery rate (FDR) of < 0.05 using the Benjamini-Hochberg (BH) test was used to assess significant enrichment of DEGs.

### Long-read sequencing analysis

#### Quality control and isoform annotation

Each sample was processed individually from subreads to annotated isoforms. Starting from subreads off of the PacBio Sequel II, ccs (v6.4.0) was used to generate high-quality, consensus (HiFi) reads consisting of concatenated cDNAs. HiFi reads were then split into S-reads (individual, barcoded cDNAs) using skera (v0.1.0). S-reads were processed using lima (v2.7.1) for proper orientation and removal of primers. This step was followed by Isoseq (v3.8.2) tag to remove the cellular barcode and unique molecular identifier (UMI) sequences and add them to the metadata for each read. Isoseq refine was used to remove polyA tails and concatemers and generated full-length, non-concatemeric (FLNC) reads, followed by isoseq correct to correct errors in cell barcode identification. FLNC reads were deduplicated using isoseq dedup, and consensus reads generated from this deduplication step were mapped to the human reference genome (GRCh38) with pbmm2 (v1.12). Mapped reads were collapsed into non-redundant isoforms using cDNA_Cupcake (v29.0) (collapse_isoforms_by_sam.py). Unique, non-redundant transcripts were then annotated using a modified version of SQANTI3^39^ and the GENCODE v44 reference annotation. SQANTI3 filtering was used to remove potential artifacts. cDNA_Cupcake’s scripts make_csv_for_dedup.py and collate_FLNC_gene_info.py were used to generate a table with reads and their associated barcodes, UMIs, gene, isoform category from SQANTI3, celltype, etc.

#### isoSeQL

Each sample was processed sequentially with isoSeQL to create an isoSeQL database unifying all the sample, isoform, and read count information. Required inputs included the filtered classification file output from SQANTI3; the genePred file from SQANTI3; the read, barcode, and cell type table from cDNA_Cupcake; and two config files including information about how the data were processed and sample metadata. Standard and custom queries were used to extract information from the isoSeQL database. isoSeQL is available at: github.com/christine-liu/isoSeQL

#### Pseudobulk DET/DTU analysis

Isoform x read count tables were generated through querying isoSeQL. DESeq2 (version 3.21) was used to identify differentially expressed transcripts (Wald test with Bonferroni correction). Only isoforms deemed “confident” through the established QC metrics were included in the analysis. DEXseq (version 1.32) was used to identify switched isoforms (generalized linear modeling with Benjamini-Hochberg adjustment). Input tables were set up such that isoforms represented features (typically exons) and grouped by gene.

#### Comparison to publicly available datasets

Fastq files from Heberle et al’s study were downloaded from Synapse with accession number: syn52047893. It was processed via pychopper to remove primer sequences and correct the orientation of the reads (double-check this). Transcripts were then mapped to the hg38 reference genome using minimap2 (nanopore parameters) and collapsed into unique, non-redundant isoforms using isoseq collapse with parameters --min-aln-coverage 0.85 --min-aln-identity 0.8 to account for the lower sequencing accuracy. Subread files (bam) from Leung et al’s study were downloaded from SRA (PRJNA664117). These subreads were processed nearly identically to our dataset. CCS generated HiFi reads; lima oriented reads and removed polyA tails; reads were mapped to the hg38 reference genome with pbmm2; transcripts were collapsed using isoseq collapse; and isoforms were annotated and filtered using our modified version of SQANTI3. Raw and processed files were made available from the PacBio Alzheimer’s Iso-Seq dataset (https://www.pacb.com/general/data-release-alzheimer-brain-isoform-sequencing-iso-seq-dataset/). We downloaded the subreads.bam and processed it identically to our dataset: generated HiFi reads using CCS, oriented read and removed polyA tails with lima, mapped reads to the reference genome with pbmm2, collapsed transcripts with isoseq collapse; and annotated and filtered isoforms using a modified version of SQANTI3. Isoforms from all three of these datasets were added to the same isoSeQL database with the rest of our samples. Isoforms from our confident set that were observed in additional samples (regardless of level or number of samples) from these other datasets were flagged.

#### Cell type assignment based on short-read analysis

Barcodes from the long-read data were matched to their corresponding cell type determined from short-read sequencing analysis. For each isoform, the % of cells of each cell type that the isoform was detected in was calculated. The isoform was determined to be expressed in a particular cell type if that cell type made up 5% or more of the total cells it was detected in.

### cDNA synthesis for RT-PCR and RT-qPCR

RNA was extracted from brain tissues using an RNeasy Plus Kit [Qiagen, 74134] according to the manufacturer’s instructions to remove genomic DNA. RNA concentrations were measured and diluted using a NanoDrop. cDNA was synthesized using SuperScript IV VILO (SSIV VILO) Master Mix with ezDNase [Invitrogen, 11766050], following the manufacturer’s instructions. Samples underwent two types of DNase treatment to ensure complete removal of genomic DNA. Final cDNA libraries were diluted to 1 ng/μl and were stored at −80 °C.

### RT-PCR confirmation of novel isoforms

PCR was performed using Platinum SuperFi II Master Mix [Invitrogen, 2368010], 10 μM primer mix, and NFW. The PCR run included the following cycle settings in the thermocycler: 98 °C for 30 seconds, then 35 cycles of: 98 °C for 10 seconds, 60 °C for 10 seconds, and 72 °C for 30 seconds, with a final extension at 72 °C for 5 minutes. PCR products and Ultra Low Range DNA ladder (10–300 bp) [Invitrogen, 10597012] or the 1 Kb plus DNA ladder (100–15000 bp) [Invitrogen, 10787018] were loaded onto a 4% or 1.5% agarose gel was prepared in 1× TAE buffer and stained with SYBR Safe DNA Gel Stain [ApexBio, A874310]. Then the gel was placed in an electrophoresis chamber and run at 100 V for 30 minutes.

#### Primer sequences:

*APOE_*NNC_FWD: AGCGGAGGTGAAGGAGCA

*APOE_*NNC_REV: CTGCATGTCTTCCACCAGG

*BIN1_*NNC_FWD: GTG AAG TTC TCG GGG AAG

*BIN1_*NNC_REV: GGAGGTGTTCTCAAGGATGAAG

*CHI3L1_*NIC_FWD: CAGATGGCAGGTCTTGCG

*CHI3L1_*NIC_REV: TCCCAACACCTGGATTTC

*CHI3L1_*FSM_FWD: CACAGCATAGTCAGTGTTGC

*CHI3L1_*FSM_REV: TTTCATGGAGCCTGGCGTG

*SEPTIN4_*FSM_FWD: GTCTGAACTCCAGGTCATCA

*SEPTIN4_*FSM_REV: GCTGCAGCCATGATCAAGC

*SEPTIN4_*NIC_FWD: ATCCAATGGCCGGAGCCCAT

*SEPTIN4_*NIC_REV: GGAGAACTGAGCAAGATGAC

### RT-qPCR confirmation of DETs

The RT–qPCR assays were performed using the CFX384 Touch Real-Time PCR Detection System [BioRad]. Reactions were performed in 10 μl triplicate solutions with 5 μl of SYBR^™^ Green Universal Master Mix [Applied Biosystems, 4309155], 1μl cDNA, 0.4 μl specific forward and reverse primer mix and 3.6 μl nuclease-free water. RT–qPCR conditions were as follows: 50 °C for 2 minutes, and 95 °C for 2 minutes following by 40 cycles of: 95 °C for 15 seconds, 58 °C for 20 seconds, and 72 °C for 30 seconds, with a final extension at 72 °C for 5 minutes. To confirm the specificity of amplification for each isoform, a melt curve analysis was performed at the end of the run. *GAPDH* was used as a housekeeping gene for normalization of each transcript’s expression levels. The relative expression and the log_2_Fold Change value were calculated using the 2^-ΔΔCt equation and log_2_(relative expression), respectively.

### Statistical analysis

Figure 1.c. Proportions of each cell type in AD and ND samples: Given the limited sample size, we employed traditional frequentist testing to assess group differences. Specifically, a two-tailed t-test was used to evaluate differences in proportion of each cell type between Alzheimer’s disease (AD, *n* = 8) and non-diseased (ND, *n* = 7) brain samples. An adjusted p-value < 0.05 was considered statistically significant.

Figure 5a. DESeq2 analysis utilized the Ward test and Bonferroni correction to determine statistically significant changes in transcript expression.

Figure 5 b–e. DEXseq analysis used linear modeling and Benjamini-Hochberg adjustment to identify isoform switching.

Figure. 6h. RT-qPCR: Expression values were log_2_-transformed prior to analysis. Differences between AD (*n* = 8) and ND (*n* = 7) brain samples were evaluated using a two-tailed Mann–Whitney U test. A p-value < 0.05 was considered statistically significant.

## Extended Data

## Supplementary Material

Supplementary Files

This is a list of supplementary les associated with this preprint. Click to download.

• SupplementaryTable7.xlsx

• SupplementaryTable5.xlsx

• SupplementaryTable1.xlsx

• SupplementaryTable2.xlsx

• SupplementaryTable6.xlsx

• SupplementaryTable4.xlsx

• SupplementaryTable8.xlsx

• SupplementaryTable3.xlsx

## Data Availability

Fastq files from short-read sequencing and bam files from long-read sequencing have been submitted to the European Genome-Phenome Archive (EGA) under accession code [will be provided in the proof process]. For privacy reasons, these data are access controlled and can be obtained after signing a Data Usage Agreement. Structure-colored isoform tracks are available at: https://genome.ucsc.edu/s/csl022/MAS_AD_confident4
